# The Challenges and Strategies towards Healthy Eating during COVID-19 Home Confinement Period among Working Adults with BMI ≥ 25 kg/m^2^ Enrolled in a Weight Loss Program: Qualitative Findings

**DOI:** 10.3390/ijerph19116656

**Published:** 2022-05-30

**Authors:** Siti Munirah Abdul Basir, Zahara Abdul Manaf, Norhayati Mohd. Noor, Arimi Fitri Mat Ludin, Suzana Shahar, Mohd Rizal Abdul Manaf

**Affiliations:** 1 Centre for Healthy Aging and Wellness and Dietetic Program, Faculty of Health Sciences, Universiti Kebangsaan Malaysia, Jalan Raja Muda Aziz, Kuala Lumpur 50300, Selangor, Malaysia; sitimunirah.abdulbasir@gmail.com (S.M.A.B.); suzana.shahar@ukm.edu.my (S.S.); 2 Center of Community Education and Wellbeing, Faculty of Education, Universiti Kebangsaan Malaysia, Bangi 43600, Selangor, Malaysia; norhayati@ukm.edu.my; 3 Centre for Healthy Aging and Wellness and Biomedical Science Program, Faculty of Health Sciences, Universiti Kebangsaan Malaysia, Jalan Raja Muda Aziz, Kuala Lumpur 50300, Selangor, Malaysia; arimifitri@ukm.edu.my; 4 Department of Community Health, Faculty of Medicine, Universiti Kebangsaan Malaysia, Jalan Yaacob Latif, Bandar Tun Razak, Cheras, Kuala Lumpur 56000, Selangor, Malaysia; mrizal@ppukm.ukm.edu.my

**Keywords:** healthy eating, challenges, strategies, COVID-19 home confinement, Social Cognitive Theory

## Abstract

The COVID-19 pandemic has been affecting our lifestyles, such as work, living, and health. In Malaysia, the Restriction of Movement Order (RMO) was first announced in March 2020 to curb the spread of the virus. Since then, many Malaysians have been confined to their own home. This new lifestyle can cause a change of eating habits where healthy eating may be a challenge. Hence, our qualitative study explored the challenges and strategies for healthy eating during the first wave of the COVID-19 home confinement period among working adults overweight and obese in Malaysia. Eleven participants were individually interviewed through phone calls. The interviews were audio-recorded, transcribed verbatim, and then coded with NVIVO 12 based on thematic analysis. We found that social pressure, changes in the social setting, more free time to access food, and extra stock of unhealthy foods at home were among the challenges to healthy eating. Some participants countered these perceived challenges by reducing unhealthy food stock, limiting kitchen visits, and utilizing self-monitoring apps to monitor their calorie intake. Social media was not consistently perceived to influence their eating behavior during this period. We conclude that COVID-19 home confinement has created challenges to healthy eating habits among overweight and obese adults with overweight and obesity. Our study provides evidence that vulnerable groups such as overweight and obese individuals require specific nutritional support during pandemic-related confinement to enhance eating self-efficacy.

## 1. Introduction

The world was shaken by the discovery of a novel coronavirus known as the severe acute respiratory system coronavirus-2 (SARS-CoV-2) in Wuhan City, Hubei Province, China in December 2019. The disease caused by this virus is known as COVID-19. The Director-General of the World Health Organization (WHO) declared this outbreak as a Public Health Emergency of International Concern (PHEC) on 30 January 2020. This outbreak was later pronounced a pandemic in March 2020 as the virus vastly spread throughout the globe. Subsequently, many countries, including Malaysia, implemented strict hygiene measures that eventually led to the Restriction of Movement Order (RMO) in Malaysia, limiting the population’s movement to break the infection chain and reduce the number of positive cases.

On 16 March 2020, the government of Malaysia announced the RMO measures involving home confinement that was initially enforced for two weeks from 18 to 31 March 2020 [1], then later extended until 28 April 2020. During this period, many activities were restricted such as grocery shopping which was required to be carried out by only one person per household, and outdoor activities were not allowed. In addition, mass movement and gatherings were prohibited, and many institutions were closed including schools, universities, and the working sectors. 

These RMO measures have changed the daily routines and lifestyles of many. Some studies have shown that mandatory confinement was associated with a negative psychological impact on adults such as stress, anxiety, exhaustion, and insomnia. Consequently, these effects combined with the experience of extensive confinement may lead to increased physical inactivity, sedentary lifestyle, and unhealthy eating patterns, for example, the increased preference for and consumption of less healthy foods and beverages [2,3,4]. These unhealthy lifestyles and poor dietary habits are among the leading causes of obesity and non-communicable diseases (NCDs), including cardiovascular diseases, cancer, and diabetes [5]. These diseases are also known as risk factors for COVID-19 mortality [6,7,8]. 

While some people struggled to ensure a sufficient supply of food, many others had difficulty cutting down their food intake to lose or maintain their weight through healthy eating during this challenging period. According to the Centers for Disease Control and Prevention (CDC), healthy eating can be defined as an eating plan that includes a variety of healthy foods prepared by a healthier cooking method such as grilling and baking to support a healthy weight [9]. It can also be defined as a calorie-restricted meal with the inclusion of essential macronutrients, micronutrients, fiber, and water [10]. Studies on the barriers to healthy eating have been widely reported. In a review by Munt et al., barriers to healthy eating include perceived lack of time, facilities, motivation, and self-regulation behaviors [11]. A workplace study among employees with obesity reported the lack of self-control and convenience and the lack of access to healthy foods as the main perceived barriers to healthy eating [12]. A great concern for the lifestyle changes caused by this pandemic is its long-term effects on body weight management in adults. Recent studies discovered that being confined at home resulted in weight gain [13,14]. In another study, a majority of its participants reported an increase in at least one weight gain-related behavior such as an inclination toward binge eating and consuming less healthy foods [15]. Higher body mass index (BMI) has been associated with lower diet quality, overeating episodes, and lower physical activity levels [16]. Given this, the Nutrition Society of Malaysia has been promoting a healthy lifestyle and tips to practice healthy eating during home confinement [17,18]. Other than that, virtual home workouts were also conducted by the Ministry of Youth and Sports to promote an active lifestyle during home confinement for the general public [19]. 

As of today, most of the dietary and lifestyle studies conducted during the COVID-19 home confinement period are quantitative cross-sectional studies conducted on the general population which include participants with BMIs ranging from normal to obese. These studies focused on changes in dietary patterns and eating behaviors [2,20,21] and information on the challenges and strategies to practice healthy eating claimed by study participants are not reported, specifically among overweight and obese adults on their weight loss journey during the confinement period. Phenomena such as experiences, attitudes, and behaviors can be difficult to accurately capture quantitatively, whereas a qualitative approach allows participants themselves to explain how, why, or what they were thinking, feeling, and experiencing at a certain time or during an event of interest [22]. Using the preliminary evidence from these studies, this qualitative study will allow a deeper understanding of the reasons that led to the changes in dietary practice and eating behaviors, and how people dealt with them during the confinement period. Lifestyle interventions targeting healthy eating behaviors are often unsustainable in the long term due to the difficulty of maintaining the changes made [22]. This unsustainability may be explained by the lack of understanding of the unique challenges faced by individuals with obesity. To note, our weight management program among overweight and obese employees (BMI ≥ 25.0 kg/m^2^) was ongoing when the first RMO was implemented. We were interested to learn about their healthy eating challenges and strategies to help us design specific strategies for the program during that period. In addition, the COVID-19 pandemic was a time of heightened stress due to unprecedented changes in our routines. Stressful adults have been shown to have higher susceptibility toward unhealthy food advertisements [23]. The rapid increase of food and drink advertising through social media during the home confinement period [24] may pose a greater impact on eating behaviors. Previous studies have shown that social media influences dietary behaviors [24,25,26]. In view of this, our current study aimed to explore the perceived challenges, the roles of social media, and the strategies used by this community in adopting and maintaining weight-related healthy eating behaviors during this challenging period. This specific community may provide the best information of interest as they already have the intention to change and have been taking action to improve their lifestyle.

## 2. Materials and Methods

### 2.1. Study Participants

We interviewed 11 working adults at a higher learning institution who participated in our research team’s workplace weight management program. Participants were volunteers from a total of 83 participants involved in the intervention. The 11 participants recruited for our interview were determined following the principle of data saturation—reaching a point where no new relevant information emerged in the interviews [27]. The program was delivered by dietitians and an exercise physiologist for 24 weeks from 5 February 2020 or six weeks before home confinement implementation until 15 July 2020. The topics covered in this program included weight-reducing diet and exercise demonstrations. Within the 24 weeks, seven face-to-face sessions were carried out at the workplace. It was then converted to an online program through a private Facebook (FB) group for another 17 sessions due to the enforcement of the RMO. The interview was carried out between sessions 9 and 12 during the online weight management intervention period, which was three weeks after the movement restriction order was enforced. The participants’ age was between 20 to 59 years with a BMI ≥ 25.0 kg/m^2^. Other inclusion criteria included healthy adults without chronic illnesses such as diabetes, heart disease, cancer, and stroke, and no history of bariatric surgery. Pregnant and breastfeeding women were excluded from this study. This study was approved by the Research Ethics Committees of the Universiti Kebangsaan Malaysia (approval code: UKM PPI.800-1/1/5/JEP-2019-391). 

### 2.2. Interview

A semi-structured interview was conducted in the Malay language through phone calls between 40 and 60 min in length between March and April 2020. All participants had given their verbal consent before the interview on top of their written consent for joining our weight management program. An interview guide was developed based on research questions guided by a literature review of relevant studies [12,28] and The Social Cognitive Theory (SCT) [29]. The interview sessions were audio-recorded using a voice recorder. The interview questions were pilot tested on two subjects before the commencement of the real interviews. 

The interviewer (SMAB) first explained the purpose of the interview followed by asking a series of specific, predetermined questions (Table 1). Three main topics were discussed: (i) the challenges towards healthy eating, (ii) the strategies used to enable healthy eating during the COVID-19 RMO period, and (iii) the role of social media on healthy eating behaviors. Participants were encouraged to share their ideas and opinions, and suitable probes were used to obtain in-depth findings. Healthy eating in this study context refers to eating based on the dietary recommendations for weight loss which includes calorie restriction and limiting sugary and fatty foods [30,31].

### 2.3. Data Coding and Analysis

The Consolidated Criteria for Reporting Qualitative Research (COREQ) framework was utilized as a guide for reporting study findings [32]. Interview recordings were transcribed verbatim. The transcripts were analyzed thematically using NVIVO^®^ 12 (NVIVO qualitative data analysis software, 2019). Each transcript was reviewed line-by-line and the identified codes; either single words or short phrases were categorized concurrently by three coders (SMAB, ZAM, and NMN). Next, the codes were grouped under broad domains of the discussion guides and theoretical constructs (e.g., social environment). Any discrepancies in coding were discussed with the research team until a mutual agreement was achieved in the final nodes as described by [33]. All researchers attempted to suspend their perspectives to avoid biases and focused on participants’ statements that described their perceptions and experiences during the interview. Findings were also presented descriptively. The complete COREQ checklist is available in the Supplementary Materials.

### 2.4. Trustworthiness

Multiple approaches were applied to ensure the trustworthiness and data quality of this study. Firstly, prolonged engagement with participants during the 6-week intervention period before the RMO helped to gain their trust and establish rapport with them. Secondly, an audit trail was used to improve the quality of the research instrument. After conducting two preliminary interviews, the lead researcher (SMAB) discussed with supervisors (ZAM and NMN) possible interview questions’ revision and probing improvement. In addition, the recordings were replayed, and the transcripts were reread to understand the essence of each interview. Lastly, continuous peer debriefing was conducted between SMAB, ZAM, and NMN to discuss data analysis and interpretation of the interviews throughout the study process.

## 3. Results

### 3.1. Participants

Data saturation was achieved after eleven Malay participants were interviewed in this study. Table 2 shows the participants’ characteristics. All of them were married and aged between 33 and 49 years. The majority of participants worked as administrative staff (73%), had tertiary education (91%), were of low to middle household income (73%), and lived with their nucleus family during the movement restriction enforcement (72%). In addition, 55% of them were within the obesity range.

### 3.2. Challenges to Healthy Eating

A total of four perceived challenges to healthy eating during the RMO were identified. The first theme was more free time to access food (food accessibility). Participants cited that they had more free time at home due to less work commitment in the early phase of RMO which eventually led to the increased frequency of snacking and cooking.

*“In the early phase of RMO, I don’t have much work to do…because I have more time at home, so I always thought about eating…I will try to ignore it at first but eventually, I will eat. I also slept late at night, around 1 am because I don’t have to rush to work in the morning. Usually, at 10.30–11.00 p.m. I’ll be hungry and have some supper. I’ll fry some kuih or cook fried noodles”*

(39-years-old female, administrative staff).

*“Before the RMO, I rarely snack at night (due to work). Now, I have time to chill with my family at night and there must be some snacking with them. I will usually have some fruits, or sometimes jam-filled buns”*

(33-years-old female, administrative staff).

Another theme that emerged from the analysis was the changes to the social environment. The social environment refers to participants’ surroundings during the RMO period. Some participants narrated that being in their respective hometowns or home confined due to the RMO changed their dietary patterns, making it more difficult for them to adhere to and maintain healthy eating habits.

*“It was harder to control my food portions in my hometown. We prepared high-calorie, high-fat foods such as curry, chicken in soy sauce, and many more. These foods were delicious, and it was difficult to turn down the temptation and control my food portion”*

(33-years-old female, administrative staff).

*“I ate more frequently in the early phase of the RMO because I cannot go out. I will usually go window shopping or a brisk walk with my family during weekends before this RMO”*

(39-years-old female, administrative staff).

Social pressure was also perceived as a challenge to sustain healthy eating habits during this period. Participants cited that their changes in eating behaviors were influenced by their family members.

*“I cook more frequently during the RMO because my kids usually ask for nuggets and sausages in between meals. So, I must cook for them. Sometimes I will join them in eating”*

(43-years-old female, academician).

*“…my wife is the one preparing food at home. During the RMO, she cooked more frequently. She’ll try new menus she’d seen on Facebook. When she cooked, I had to eat to please her”*

(37-years-old male, administrative staff).

Lastly, extra unhealthy food stock (food availability) was identified as another challenge to healthy eating during this period for some participants. Some participants claimed that there was an increased availability of unhealthy, processed food stocks at home as compared to the pre-COVID-19 period. This factor consequently increased their cooking and eating frequency during the COVID-19 home confinement period.

*“…I can say 50% of the food bought were processed foods such as instant noodles, nuggets, burger patties…they are easy to prepare. So, when there’s stock, we’ll cook and eat them. (As a result, my eating frequency has increased from three to five or six times a day”*

(36-years-old male, administrative staff).

*“We have a constant supply of milk chocolate and nuts at home…I also noticed that I love snacking on nuts during this time. Sometimes I snacked on nuts with my kids and usually, I consumed about half of it”*

(43-years-old female, academician).

### 3.3. Strategies to Practice Healthy Eating

The discussion on the strategies to practice healthy eating during the RMO period with the participants yielded two themes: self-control and self-monitoring. Under the self-control theme, seven participants tried to control their food intake either by limiting their purchases of snacks, reducing kitchen visits, fasting, or committing to food portion control.

*“…in terms of snacking, I avoid buying snack foods because I would consume them if they were available in the house”*

(37-years-old male, administrative staff).

*“I avoid going to the kitchen after 3 p.m. No kitchen visit after lunchtime”*

(38-years-old female, administrative staff).

*“Fasting is my only way to control my diet intake”*

(33-years-old female, administrative staff).

*“I do portion control. Since I was alone, I will cook just enough for myself. Other than that, I will limit the use of oil in my cooking”*

(45-years-old female, administrative staff).

Other participants mentioned the use of self-monitoring apps to help them in monitoring their daily calorie intake.

*“The MyFitnessPal app helps me to monitor the calories in my food intake…I know how to adjust my intake based on my calorie limit”*

(49-years-old female, administrative staff).

*“I am using MyFitnessPal to ensure my calorie intake is within 2200 kcal/day. If I have exceeded the limit, I will make sure to counter it with extra exercise”*

(37-years-old male, administrative staff).

*“In terms of quantity, I will ensure my daily intake is within 1500 kcal/day. I have a monitoring app on my phone”*

(36-years-old male, administrative staff).

### 3.4. Roles of Social Media

The discussion on the role of social media in healthy eating behavior with the participants highlighted three main themes. The first theme identified social media as a negative influence. Some participants mentioned that social media changed their dietary patterns due to food ideas and temptations from social media.

*“During the early phase of RMO, I ate more frequently compared to before…I and my wife will scroll our social media and try new recipes shared by others”*

(36-years-old male, administrative staff).

*“My wife likes to try new menus shared by her friends. I consume what was prepared by my wife. So, somehow it does change my diet”*

(37-years-old male, administrative staff).

Another theme identified was social media as a positive influence. Some participants viewed social media as a source of social support and motivation to maintain healthy eating habits. In other words, it provides positive behavioral enhancement through the support and positive examples from their peers.

*“Sometimes my friends shared the healthy foods and exercises they’ve made through the WhatsApp group. I’ll try to follow what they did”*

(37-years- old male, administrative staff).

*“The WhatsApp group is one of the reasons I am back on track (for healthy eating). The reminders and tips shared act as a motivator for me to not give up”*

(36-years-old male, administrative staff).

Lastly, seven out of eleven participants claimed that social media did not influence their dietary habits and food choices during the RMO (neutral).

*“In my opinion, social media does not influence my diet. It is not our practice to add new menus to our daily meals. If we are going to try a new menu, then we will swap it with any of the main meals of the day”*

(32-years-old male, administrative staff).

*“I am rarely active on social media except for WhatsApp. I am not influenced by any trends during the RMO”*

(46-years-old male, academician).

*“Honestly, they (social media) did not influence my diet during RMO. I am not interested in all the food trends”*

(33-years-old female, administrative staff).

**Figure 1:**
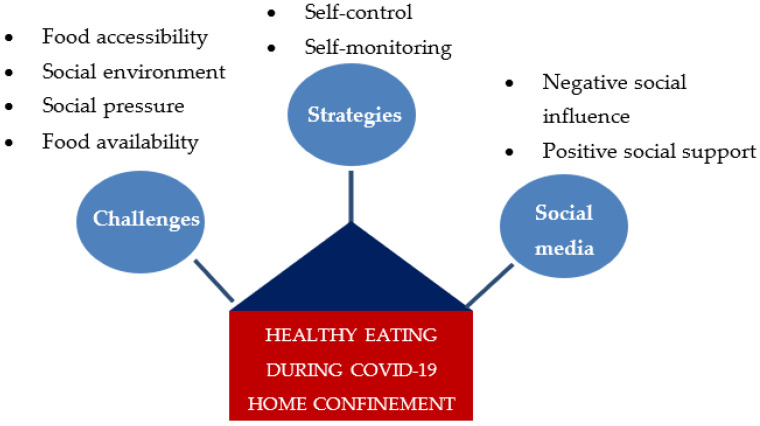
The challenges and strategies to healthy eating, and the roles of social media.

Figure 1 summarizes the challenges to healthy eating during the RMO home confinement period and the strategies employed to sustain it, and the roles of social media as determinants of healthy eating behaviors.

## 4. Discussion

This study provides an insight into the perceived challenges imposed by COVID-19 home confinement on healthy eating behaviors among employees who are overweight and/or obese and their strategies for overcoming those challenges. Other than that, we explored the role of social media in healthy eating behaviors during this period. 

Being confined at home creates a challenging environment for healthy eating. Our study found that home confinement provided extra free time for the participants to have access to food. Before the pandemic, these participants would usually be busy at work during the day which limited their time for eating, snacking, or cooking. However, their workload was reduced during the RMO home confinement period. Furthermore, later bedtime during this period provides more free time at night. These may have led to boredom which triggered their thoughts on foods and eventually, resulted in increased snacking and cooking frequency. Previous studies suggested that boredom is one of the predictors of eating behavior [34,35]. Our study did not investigate the emotional factors that contributed to increased food intake. COVID-19 home confinement may be a necessary precaution to curb the spread of the virus, but it has negatively affected the emotional and psychological states of the population. The increase in depressed mood and anxious feelings among general populations during this period was widely reported [36,37,38]. A study among 602 Italian adults found that over-eating was associated with anxious feelings during the COVID-19 home confinement period [2]. It was found that more than half of them increased their food consumption to feel better. This shows that psychological wellbeing may also play a role in the increased frequency of snacking and cooking in this challenging period. Contrary to our findings, the lack of time to plan, shop, as well as prepare and cook healthy foods were reported as a barrier to healthy eating in similar past studies [11,39,40]. Home confinement could be an opportunity to increase healthy eating behavior as individuals have ample time to plan their meals appropriately.

We also identified the perceived change in the social environment as one of the factors that affected healthy eating behaviors among the participants. One participant reported consuming rice more frequently during his main mealtime as compared to before the RMO period because it was a norm back in his hometown. In addition, participants’ food choices had also changed due to the different food preparation methods which were mostly high in calories, fat, sugar, and salt. These participants were more likely to have little control over food preparation decisions as they have to follow the preferences of their families. This finding is in line with a recent review where an unhealthy diet among family units was reported as one of the barriers to healthy eating [11]. Apart from this, home confinement also limited physical mobility to curb the spread of COVID-19. Consequently, participants’ life routines such as outdoor recreational activities were halted. In response to this, some participants filled the void with eating and, as a result, their food intake increased. Furthermore, eating may be a way to cope with psychological distress for these participants. The inability to carry out usual pre-pandemic activities could have affected their mental health. Numerous studies reported that mental health disorders such as depression, anxiety, and stress have escalated during COVID-19 home confinement [3,41]. A study among Malaysian couples found significantly higher levels of perceived depression, anxiety, and stress during the RMO compared with before the RMO [42]. These negative effects have been associated with negative eating behaviors such as overeating and increased meal frequency [43,44].

Another theme identified as a challenge towards healthy eating was perceived social pressure. This pressure came from the family members of the participants such as their spouses and children. As for the male participants, they had little control over food preparations at home. Culturally, it is women’s responsibility to cook for their families, which includes the selection, purchasing, processing, and production of food at home [45]. Male participants in this study reported that they felt pressured to consume the meals prepared by their wives to show their appreciation for their cooking. Regarding the female participants, they reported that they cooked more frequently to attend to their children’s requests. Our finding is supported by previous studies which have cited that pressure from the social circle can act as a challenge or barrier to healthy eating [46,47]. Increased cooking frequency consequently can cause an increase in daily food intake. Similarly, recent studies among adults during social lockdowns revealed that cooking [4] and eating frequency increased during this period [4,14,16,48,49].

Stockpiling and panic buying were reported as common behaviors when home confinement or lockdown was first announced by the government around the world [50], including Malaysia. Stockpiling is a desire to minimize the loss of access to resources by accumulating goods in response to a belief of product scarcity [51]. It is a fear-based behavior in response to the shortage of goods. From this study, it was found that participants agree that increased stockpiling of unhealthy foods such as burger patties, nuggets, and instant noodles was one of the perceived challenges to adhering to healthy eating habits during the RMO home confinement. They perceived an increase in cooking and eating frequency due to this factor. In the early implementation of RMO in Malaysia, a lot of activities were restricted including grocery shopping. A limited number of shoppers were allowed at a shopping premise at one time, causing them to spend extra grocery hours due to the long queues. Hence, stockpiling is a way for them to reduce the frequency of grocery shopping. A recent study has shown that the amount of high-calorie snack foods, desserts, and non-perishable processed foods purchased had increased during the COVID-19 pandemic [41,52]. Stockpiling on ready-to-cook and ready-to-eat foods which are commonly high in calories and fats could pose a great risk to weight gain during this period.

Despite the challenges, participants perceived healthy eating behaviors were manageable through two main strategies which were self-control and self-monitoring. Self-control in the context of healthy eating behaviors refers to one’s ability to overcome unhealthy food consumption and adopt healthier behaviors to achieve their healthy eating goal [53]. It plays a vital role in healthy eating adherence in an environment that encourages unhealthy eating behavior [54]. Our study found that self-control approaches used by our study participants include refraining from buying snacks and limiting kitchen visits. These actions were perceived to help them in managing their daily calorie intake and avoiding unnecessary snacking. Studies have shown that a high level of self-control is associated with better adherence to healthy eating habits and fewer bingeing episodes [55,56]. In addition, lower self-control capability is associated with increased body weight and obesity risk. Thus, self-control is an important attribute in weight management. Fasting was also mentioned as a way to control food intake during this home confinement period. Self-control is the essence of fasting as it involves abstinence from eating and drinking for a certain stretch of time [57]. Uncontrolled food intake and weight gain could be of concern among those aiming for weight loss. Fasting is one of the aids to weight loss as it involves spontaneous caloric restriction [58]. Furthermore, this practice has been shown to aid in internal control over negative eating behaviors such as overeating and consumption of unhealthy foods [59].

Self-monitoring is also an important agent for behavioral change including dietary changes [60] and weight loss [61,62]. It increases one’s attention and adherence to target behavioral change [63]. About half of our study participants used a self-monitoring mobile application to monitor their food and calorie intake. There are many self-monitoring apps related to dietary and weight management available in the online market. These apps are most popular because they help individuals to improve their health behaviors via self-monitoring [64,65]. These apps were found to be helpful among our study participants in monitoring their calorie intake and to plan their daily meals. Previous studies have also shown that self-monitoring mobile apps improve dietary behaviors [66,67]. They are helpful in increasing motivation and desire to achieve goals, improving an individual’s self-efficacy, and increasing the frequency and consistency of eating healthy foods [68]. In addition, logging food intake into the apps may increase self-awareness, consequently improving healthy food consumption [69]. Social media is a platform used by many to share their thoughts, ideas, and even lifestyle. To date, over half of the world population (58.4%) uses social media with an average usage of 187 min per day. Recently, it was reported that the Internet traffic was 23.5% higher in the first week of COVID-19 home confinement and increased further by another 8.6% in the following week [70]. In the MCMC’s latest survey, it was found that the majority of Internet users preferred going online on their social media platforms and its use has increased by 7% from 2018 to 2020 [71]. Previous studies have shown that social media plays a role in determining individuals’ dietary behaviors and perceptions of their nutrition and physical activity [24,25]. For example, a study among adults aged 18 years and above indicated that social media platforms such as Facebook positively influenced individuals’ nutritional approaches and attitudes, encouraging them to improve their eating habits [25]. In our study, some participants claimed that social media influenced their food choices. They had the tendency to try out new recipes or foods that they encountered while scrolling through social media platforms. Our findings show that social media plays a role in determining users’ eating behaviors. This finding is supported by a recent study by Hawkins and colleagues where they found that perceived norms of social media users’ eating habits predict participants’ consumption of fruits and vegetables, energy-dense snacks, and sugar-sweetened beverages (SSB) [26]. Despite its influence on negative eating habits, social media was also used as a platform for social support in maintaining healthy eating habits among our study participants. Previous studies have shown that support from surrounding people such as family members and friends is one of the key determinants of healthy eating behaviors [72,73,74].

Reciprocal determinism in the Social Cognitive Theory (SCT) describes the interaction between personal, environmental, and behavioral factors in behavioral changes and how they influence each other [75]. The COVID-19 home confinement order had changed the participants’ physical and social environment. An individual’s physical surroundings and social factors may act as facilitators or barriers to behavioral changes [76]. In reference to our study, efforts toward healthy eating practices were impeded by food accessibility and availability (physical factors), the food preferences of family members, and social media (social factors). As described in the SCT, the precondition for health-related behavioral change is the knowledge of the health risk and benefits [76]. In our study, participants have been exposed to weight management education which facilitated their self-control and self-monitoring behaviors in maintaining healthy eating habits during this challenging period.

This study adds to the growing literature on dietary behaviors during pandemic-related home confinement, specifically among overweight and obese working adults undergoing a weight loss intervention program. We have highlighted the role of time, the social environment, social pressure, and food stockpiling as the challenges to healthy eating behaviors among this population during this period. As our study also highlights the importance of self-control and self-monitoring in behavioral change, future studies may consider enhancing these abilities in weight management programs, particularly during the pandemic-related home confinement period.

### 4.1. Limitations of the Study

Despite its strengths, this is an institution-based study among employees who are overweight and obese with a majority representation from an administrative group. Hence, our findings only reflect the perceptions of this group. As our study participants were all Malay and married, the findings do not demonstrate the opinions of other ethnicities and unmarried staff. Due to several technological limitations during this challenging home confinement period such as poor internet coverage, triangulation with focus group discussions could not be done to support our findings. Our study participants are employees with a BMI ≥ 25.0 kg/m^2^ who were undergoing a weight management program during the COVID-19 home confinement period. Therefore, our research findings may not be generalizable to the rest of the population due to the small sample size and sociodemographic boundaries. However, it should be noted that data saturation has been achieved within this small sample size. The aim of our study is not to generalize findings; but rather, to develop a rich understanding of the subject matter among our study participants during the home confinement due to the pandemic.

These limitations warrant further exploration among adults with a normal BMI who have been through a similar home confinement experience. Furthermore, weight management intervention programs during pandemic-related home confinement should focus on empowering healthy eating behaviors by tackling the challenges participants face at home.

### 4.2. Practical Implications

Our findings serve as important evidence that pandemic-related environmental changes lead to new eating challenges among adults in a weight loss program. The new challenges may affect an individual’s eating self-efficacy and consequently lead to weight loss failure. It is crucial for healthcare professionals to assess and acknowledge this issue not only to enhance weight management approaches but also to improve the community’s lifestyle in general by being equipped with the necessary tools and knowledge of the newly distinguished field of lifestyle medicine such as the Healthy Lifestyle and Personal Control Questionnaire (HLPCQ) [77,78,79].

## 5. Conclusions

In summary, free time for food access, the social environment, social pressure, and unhealthy food stockpiling are the perceived challenges to healthy eating among overweight and obese working adults during COVID-19 home confinement. The adoption of healthy eating habits has been facilitated by perceived self-control measures such as reducing unhealthy food stockpiling, limiting kitchen visits, and self-monitoring their calorie intake by utilizing self-monitoring apps. In addition, social media was perceived to have positive and negative influences on behavioral changes toward healthy eating. In conclusion, overweight and obese adults undergoing a weight management program perceived COVID-19 home confinement as a challenging period to sustain healthy eating behaviors Our study findings suggest that facilitating self-control and self-efficacy in weight-related eating behaviors are imperative during the challenging pandemic-related home confinement period. Tailoring community-based approaches by considering the eating challenges in a restricted new environment such as this pandemic can help interventionists and their participants/clients better achieve their goals.

## Figures and Tables

**Table 1:** Interview questions guide.

Topic	Questions
Challenges	(1) Did your dietary intake change during the RMO home confinement? If yes, please explain what the changes are. (2) What do you think caused the changes?
Strategies	What are your strategies to monitor your diet during the RMO?
Role of social media	Did social media (e.g., WhatsApp, Facebook & Instagram, etc.) influence your diet during the RMO? If yes, can you explain how it influenced your diet?

**Table 2:** Participants’ characteristics.

Characteristics	*n*	(%)	Mean	SD
Gender				
Male	5	45		
Female	6	55		
Age			39.6	5.4
30–39 years	7	64		
40–49 years	4	36		
Marital status				
Married	11	100		
Education				
Secondary school	1	9		
Tertiary education	10	91		
Occupation				
Administrative staff	9	82		
Academician	2	18		
Household Monthly Income (USD *)				
Low (<1144)	3	27		
Middle (1145–2546)	5	46		
High (>2546)	3	27		
Living arrangement				
Alone	1	9		
With nucleus family	8	73		
With extended family	2	18		
BMI (kg/m^2^)			34.1	6.9
25.0–29.9	5	45		
≥30.0	6	55		

* Categorization based on Bottom 40%, Middle 40%, and Top 20% income group.

## Data Availability

All relevant information is within the manuscript and Supplementary Materials file.

## Supplementary Materials

The following supporting information can be downloaded at: https://www.mdpi.com/article/10.3390/ijerph19116656/s1, Supplementary Materials File S1: COREQ checklist.
