# Supplementary material for: The Challenges and Strategies towards Healthy Eating during COVID-19 Home Confinement Period among Working Adults with BMI ≥ 25 kg/m2 Enrolled in a Weight Loss Program: Qualitative Findings
Source: Int J Environ Res Public Health. 2022 May 30;19(11):6656. doi: 10.3390/ijerph19116656 (PMC9180068; doi:10.3390/ijerph19116656)
Supplement: Supplementary file 1 [file ijerph-19-06656-s001.zip › ijerph-1697389-supplementary.pdf]

**Supplementary Materials File S1: COREQ checklist**<br>**Domain 1: Research team and reflexivity**	**Location in the manuscript (section, page no.)**	
**Personal characteristics**		
1. Interviewer/facilitator<br><br>Which author/s conducted the interview or focus group?	Siti Munirah Abdul Basir (SMAB)	
2. Credentials<br><br>What were the researchers' credentials? E.g. PhD, MD	MHSc, PhD	Title page - 1
3. Occupation<br><br>What was their occupation at the time of study?	Researcher and lecturer	
4. Gender<br><br>Was the researcher male or female?	Female	
5. Experience and training<br><br>What experience or training does the researcher have?	The researcher (SMAB) was in the middle of completing her Ph.D. where principles of qualitative research were covered in detail under experienced qualitative researcher (NMN) supervision.	Methods – page 6 & 7
**Relationship with participants**		
6. Relationship established<br><br>Was a relationship established prior to study commencement?	Yes	Methods – page 7
7. Participant knowledge of the interviewer.<br><br>What did the participants know about the researcher? E.g. personal goals, reasons for doing the research.	Participants were briefed on the purpose of the study and understood that it was a research project for the researcher (SMAB). Ethical approval had been granted, and participants had given their verbal consent on top of their written consent for joining	Methods – page 5

	our weight management program.	
8. Interviewer characteristics<br><br>What characteristics were reported about the interviewer/facilitator? E.g. bias, assumptions, reasons, and interest in the research topic.	Researcher (SMAB) is a full time PhD student. No other interviewer-related biases identified.	Methods -
**Domain 2: study design**		
**Theoretical framework**		
9. Methodological orientation and theory<br><br>What methodological orientation was stated to underpin the study? E.g. grounded theory, discourse analysis, ethnography, phenomenology, content analysis.	Thematic content analysis.	Methods – page 6
**Participant selection**		
10. Sampling<br><br>How were the participants selected? E.g. purposive, convenience, consecutive, snowball.	Participants derived from the recent study, purposive sampling.	Methods – page 5
11. Method of approach<br><br>How were the participants approached? E.g. e.g. face-to-face, telephone, mail, email.	Telephone	Methods – page 5
12. Sample size<br><br>How many participants were in the study?	Eleven	Methods – page 4
13. Non-participation<br><br>How many people refused to participate or dropped out? Reason?	None	
**Setting**		
14. Setting of data collection<br><br>Where was the data collected? E.g. home, clinic, workplace.	Data were collected at home via phone calls.	Methods – page 5
15. Presence of non-participants	No.	

Was anyone else present besides the participants and researchers?		

16. Description of sample<br><br>What are the important characteristics of the sample? E.g. demographic data, age.	Participated in our weight management program and working from home during Movement Control Order (MCO).	Methods – page 4
**Data collection**		
17. Interview guide<br><br>Were questions, prompts, guides provided by the authors? Was it pilot-tested?	Interviews were semi-structured and follow-up questions were allowed.	Table 1 – page 6
18. Repeat interviews<br><br>Were repeat interviews carried out? If yes, how many?	No.	
19. Audio/visual recording<br><br>Did the research use audio or visual recording to collect the data?	Yes, interviews were audio-recorded.	Methods – page 5
20. Field notes<br><br>Were field notes made during and/or after the interview or focus group?	Yes.	Methods - Page 5
21. Duration<br><br>What was the duration of the interviews or focus group?	40-60 minutes	Methods – page 5
22. Data saturation<br><br>Was data saturation discussed?	Yes	Methods – page 6
23. Transcripts returned<br><br>Were transcripts returned to participants for comment and/or correction?	No.	

Domain 3: analysis and findings		
24. Number of data coders<br><br>How many data coders coded the data?	Three	Methods – page 6
25. Description of the coding tree.<br><br>Did author provide a description of the coding tree?	Yes	Methods – page 6
26. Derivation of themes<br><br>Were themes identified in advance or derived from the data?	Themes were derived from the data.	Methods
27. Software<br><br>What software, if applicable, was used to manage the data?	NVivo 12 plus	Methods – page 6
28. Participant checking<br><br>Did participants provide feedback on the findings?	No.	
**Reporting**		
29. Quotations presented<br><br>Were participant quotations presented to illustrate the themes/findings? Was each quotation identified? E.g. participant number.	Yes, specific comments were supported with direct quotes attributed to an anonymized participant by their age and occupational group.	Results – Table 3 – 5
30. Data and findings consistent<br><br>Was there consistency between the data presented and the findings?	Yes	Discussion – page 14-18
31. Clarity of major themes<br><br>Were major themes clearly presented in the findings?	Yes	Results – Table 3-5

32. Clarity of minor themes<br><br>Is there a description of diverse cases or discussion of minor themes?	No.	
